# The Efficacy of the Naples Prognostic Score in Patients with Carotid Stenosis

**DOI:** 10.1055/s-0046-1817033

**Published:** 2026-04-07

**Authors:** Derya Ozdogru, Mehmet Necmi Eke, Ilker Ozturk, Pamir Bastin, Onur Kadir Uysal, Akkan Avci, Zulfikar Arlier

**Affiliations:** 1 University of Health Sciences, Adana City Research and Training Hospital, Department of Neurology, Adana, Türkiye.; 2 University of Health Sciences, Adana City Research and Training Hospital, Department of Cardiology, Adana, Türkiye.; 3 University of Health Sciences, Adana City Research and Training Hospital, Department of Emergency Medicine, Adana, Türkiye.

**Keywords:** Carotid Stenosis, Ischemic Stroke, Inflammation, Nutritional Status

## Abstract

**Background:**

The Naples Prognostic Score (NPS) is widely used, especially in cancer patients. Although it has been emphasized that this scoring system has a strong prognostic power for cardiovascular diseases, studies on cerebrovascular patients are quite limited.

**Objective:**

To investigate whether there is a relationship between the level of carotid artery stenosis and the NPS.

**Methods:**

Measurements were taken and recorded using venous blood samples taken from the patients at the time of their first hospital admission. Patients with NPS of 0, 1, and 2 were classified as the *low Naples group* . Patients with NPS of 3 and 4 were classified as the *high Naples group* . When analyzing the patients, comparisons were made according to these two separate groups.

**Results:**

According to the cut-off values of the neutrophil-to-lymphocyte ratio (NLR; > 2.96), the lymphocyte-to-monocyte ratio (LMR; < 4.44), total cholesterol (TC; < 180), and albumin (< 4), the NLR was high in 261 (40%) patients, the LMR was low in 113 (17.3%) subjects, the TG level was low in 215 (32.9%) participants, and the albumin level was low in 247 (48.4%) individuals. The NPS was calculated according to the sum of the NLR (> 2.96), LMR (< 4.44), TG (< 180) and albumin (< 4) cut-off values, and it was 0 in 17 (2.6%) patients, 1 in 87 (13.3%), 2 in 212 (32.5%), 3 in 214 (32.8%), and 4 in 123 (18.8%) subjects.

**Conclusion:**

The current study revealed a significant correlation between the degree of carotid stenosis and the NPS, which can be used as a tool to predict the severity of carotid stenosis.

## INTRODUCTION

Acute ischemic stroke affects approximately 87% of all stroke patients, and it is recognized that the underlying cause in nearly all of these cases is thrombosis or embolism. 1 Moreover, it is recognized that the underlying cause of approximately 10 to 15% of these ischemic strokes is atherosclerotic carotid stenosis. 2 Acute ischemic stroke represents a substantial global health concern, contributing significantly to mortality rates. 3 Beyond its high mortality rate, it is recognized as a significant cause of chronic sequelae and widespread disabilities in survivors. 4 5 6 Given the high mortality and morbidity rates, there is a strong need to identify prognostic markers in diagnosis and to perform emergency interventions.

It is imperative to acknowledge the pivotal role of inflammation in the pathophysiology of acute ischemic stroke. 7 Some studies 8 9 10 11 have indicated that parameters such as the neutrophil-to-lymphocyte ratio (NLR), the lymphocyte-to-monocyte ratio (LMR), the monocyte-to-high-density lipoprotein ratio (MHR), the levels of C-reactive protein (CRP), and the fibrinogen-to-albumin ratio (FAR) have a strong prognostic effect.

In recent years, the combination of nutritional status evaluation and inflammatory parameter scoring has emerged as a novel approach in stroke research. A notable example of such a system is the Naples Prognostic Score (NPS), which has gained widespread application, particularly in the context of cancer patients. 12 While the system has been lauded for its substantial prognostic capabilities in the context of cardiovascular diseases, there is a paucity of research specifically focused on cerebrovascular patients. 7 12 13 14 15 To our knowledge, no study has examined the relationship between the level of carotid artery stenosis and the NPS using digital subtraction angiography (DSA). Given that the degree of carotid stenosis may influence the risk of thromboembolism, we believe it is important to investigate this relationship. Therefore, the purpose of the current study was to investigate the relationship between the level of carotid artery stenosis and the NPS.

## METHODS

### Patient selection and data collection

The present was conceived as a retrospective cross-sectional study. Prior to the initiation of the study, ethical approval was obtained from the Adana City Training and Research Hospital's Scientific Research Ethics Committee (date: August 15, 2024; meeting number: 4; decision number: 107). Data on patients who sought treatment at the stroke clinic between April 1st, 2019, and July 31st, 2024, and who met the study criteria were obtained from the hospital automation information system. All biochemical parameters were obtained from blood samples taken during outpatient evaluation, regardless of whether the patients were admitted for interventional procedures or not. Patients with low-grade carotid artery stenosis were not hospitalized but were evaluated on an outpatient basis. Patients with high-grade carotid artery stenosis were hospitalized and underwent DSA. The final study population included 653 patients. The first group comprised patients with > 70% of carotid stenosis, as determined by DSA. The second group comprised patients with 50 to 69% of carotid stenosis, and the third group comprised patients with < 50% of carotid stenosis. Patients older than 18 years were included, and those under the age of 18 whose file data could not be fully accessed were excluded. Patients with trauma, infectious pathologies, malignancy, those morbidly obese and presenting other inflammatory diseases were also excluded. The demographic data of the patients were recorded from their files.

### Laboratory analysis and calculation of the NPS

The measurement data were calculated and recorded using venous blood samples taken from the patients at the time of their admission to the outpatient clinic. The following parameters were measured: the levels of neutrophils, lymphocytes, platelets, hemoglobin, and C-reactive protein, as well as renal function tests, liver function tests, serum lipid parameters, and albumin levels. The measurements were taken using the UniCel DxC 800 Syncron autoanalyzer (Beckman Coulter Inc.). The NPS was calculated based on four parameters: the NLR, the LMR, the total cholesterol level, and the albumin level. The NLR value was assigned 1 point if it exceeded 2.96, the LMR, if it equaled or was lower than 4.44, the total cholesterol level, if it was ≤ 180 mg/dL, and the albumin level, if it was lower than 4.0 mg/dL. 12 Patients with NPS of 0, 1, and 2 were classified as the *low Naples group* , and those with NPS of 3 and 4, as the *high Naples group* ; when analyzing the patients, comparisons were made according to these two groups.

### DSA application procedure

Patients who applied to the stroke clinic and had < 50% internal carotid artery (ICA) stenosis in the images after cervical computed tomography (CT) angiography or magnetic resonance (MR) angiography were included in the study as the third group of patients. Patients in the first and second groups had > 50% ICA stenosis, and cerebral DSA was performed on them. The procedure was performed with an Artis zee, model floor, C-arm monoplane angiography device (Siemens Healthineers AG). The area designated for puncture was meticulously cleansed with povidone-iodine (Batikon), followed by the application of 10 mg of local lidocaine (Jetocaine). Access was then gained from the right groin using a 5F femoral catheter sheath. Aortography was then performed with a pigtail catheter placed after the guide wire. Subsequently, right and left carotid and vertebral system angiography images were obtained using one or more of the 5F Vertebral, 6F Judkins, 5F Simmons type-I, and 5F Simmons type-II catheters. The selection of the catheter was informed by the arch type. The degree of stenosis was determined by the North American Symptomatic Carotid Endarterectomy Trial (NASCET) method.

### Statistical analysis

The statistical analysis was conducted using the IBM SPSS Statistics for Windows (IBM Corp.) software, version 25.0. The categorical measurements were expressed as numbers and percentages, and the continuous measurements, as mean and standard deviation values (or median and minimum–maximum values when necessary). The Kolmogorov-Smirnov test was employed to ascertain the distribution of the parameters within the study sample, and the Mann-Whitney U test was used in paired group analyses for parameters that did not show normal distribution. The Chi-squared test was employed to perform comparisons between categorical expressions. The statistical significance level was set at 0.05 in all tests.

## RESULTS

Of the patients, 243 (37.2%) were female and 410 (62.8%) were male subjects, with a mean age of 67.8 ± 9.2 (median: 69) years. Furthermore, data analysis revealed that 227 (34.8%) patients presented a history of smoking. The prevalence of additional diseases was as follows: hypertension (HT) in 544 (83.3%), diabetes mellitus (DM) in 312 (47.8%), coronary artery disease (CAD) in 342 (52.4%), bypass in 95 (14.5%), congestive heart failure (CHF) in 32 (4.9%), chronic obstructive pulmonary disease (COPD) in 82 (12.6%), chronic kidney disease (CKD) in 90 (13.8%), and hyperlipidemia in 279 (42.7%). Of the patients, 172 (26.4%) had stenosis with a score below 50%, 379 (58%) had unilateral stenosis, and 102 (15.6%) had bilateral stenosis ( Table 1 ).

Furthermore, 84 subjects (12.9%) exhibited stenosis scores between 50 and 69%, 397 patients (60.8%) demonstrated stenosis scores below 70%, and 172 subjects (26.3%) were in the control group.

According to the established cut-off values for the NLR (> 2.96), LMR (< 4.44), total cholesterol (< 180 mg/dL), and albumin (< 4 mg/dL), the following proportions of patients were observed: 261 (40%) had high NLR, 113 (17.3%) had low LMR, 215 (32.9%) had low total cholesterol, and 247 (48.4%) had low albumin.

The NPS, a composite metric derived from the sum of the cut-off values of the NLR, LMR, total cholesterol, and albumin, was assigned as follows: 0 in 17 (2.6%), 1 in 87 (13.3%), 2 in 212 (32.5%), 3 in 214 (32.8%), and 4 in 123 (18.8%) ( Table 1 ).

Among the patients with elevated NPS, the male-to-female ratio and the prevalence of HT and COPD were found to be significantly higher ( *p* < 0.001, *p* = 0.033, and *p* = 0.044 respectively). Conversely, patients with high NPS exhibited a lower prevalence of < 50% of stenosis ( *p* = 0.003) ( Table 2 ).

Furthermore, the mean age and height were found to be elevated ( *p* = 0.001 and *p* < 0.001 respectively), while the mean Body Mass Index (BMI) value was found to be reduced ( *p* = 0.002). Moreover, the analysis revealed that patients with high NPS exhibited significantly-elevated mean levels of neutrophils, creatinine, blood urea nitrogen, and NLR ( *p* < 0.05). Concurrently, these patients demonstrated reduced mean levels of lymphocytes, monocytes, hemoglobin, hematocrit, platelets, glomerular filtration rate, albumin, total cholesterol, high-density lipoprotein, low-density lipoprotein, and LMR ( *p* < 0.05) ( Table 2 ).

## DISCUSSION

The current study is pioneering in its examination of the relationship between the NPS and carotid stenosis, and in demonstrating its correlation with the degree of stenosis. A salient finding is that the proportion of patients who underwent DSA and were found to have carotid stenosis above 70% was significantly higher for the group with a high NPS. Conversely, the NPS was found to be low in patients with carotid stenosis below 50%. This finding underscores the efficacy of the NPS as a robust and independent predictor of carotid stenosis in DSA. A recently-published retrospective analysis 16 of 697 patients who underwent carotid artery stents revealed a significant correlation between the mortality rates of these patients and high NPS after the long-term follow-up. However, it is important to note that this study exclusively focused on patients who underwent carotid artery stent treatment, which may limit the generalizability of its findings. The relationship between carotid artery stenosis and the NPS remains to be fully elucidated. In this context, the results of the present study are of particular significance, as they are the first of their kind.

A significant body of research 17 has identified the critical role of inflammatory parameters in disease development. It is well known that these inflammatory processes play a role in a wide variety of cardiovascular diseases and conditions involving atherosclerosis. In the context of acute ischemic events, the onset of inflammation in the ischemic region occurs within the first 24 hours. It is noteworthy that this inflammation can further exacerbate ischemic damage. 7 Reduced cerebral blood flow has been shown to trigger inflammation. The release of intravascular leukocytes from the vascular endothelium, combined with the activation of proinflammatory mediators, contributes to the exacerbation of neurodegenerative effects in brain tissue. Consequently, an inflammatory cascade response is elicited. 18 19 Activation of inflammation leads to the migration of neutrophils across the blood-brain barrier, resulting in rapid neurodegeneration in the first hours following stroke. 20 This has the potential to exacerbate brain injury and lead to hemorrhagic transformation in ischemic strokes. 21 Unlike neutrophils, some lymphocytes are known to exhibit a neuroprotective function. 22 It is crucial for clinicians to ensure that the neuroprotective and neurodegenerative balance is appropriate for the patient and the neuroprotective process. In cases of significantly-increased carotid stenosis, inflammatory markers such as the NLR and LMR are of paramount importance, especially given the possibility of acute ischemic stroke. The results of the current study demonstrate that patients with high NPS exhibit high NLR and low LMR. These findings highlight the critical importance of the NLR and LMR in determining the prognosis of patients with carotid stenosis and emphasize the need for clinicians to incorporate these parameters into their clinical assessments and treatment strategies.

It is well known that albumin plays a significant role in the development and progression of thrombosis. 23 When the nutritional status of the patients deteriorates, the liver is induced to increase albumin synthesis while simultaneously producing clotting factors. This leads to a state of hypercoagulability, which can lead to increased blood viscosity and an increased risk of thrombosis. 24 25 Furthermore, lipids have been shown to play a significant role in the inflammatory response. It has been hypothesized that the inflammatory process, particularly in thromboembolic events, progresses through a multifaceted process affecting cholesterol synthesis and the transport and absorption of hepatic lipids. Several studies 25 26 have demonstrated the relationship involving cholesterol levels and short-term prognosis and recurrence of thromboembolism. In the present study, we found that albumin levels were lower and total cholesterol levels were higher in patients with higher NPS. Although this suggests that the NPS may be a useful tool for clinicians to consider in estimating the risk of thromboembolism, more longitudinal data are needed to confirm the association between this score and clinical outcomes and to determine its prognostic significance in patients with carotid disease.

One of the important results of the current study is the statistically significant lower BMI in patients with a high NPS. Low BMI/underweight is widely recognized as a global indicator of malnutrition associated with adverse outcomes. 27 Furthermore, obtaining BMI simultaneously with a history of weight loss will help improve practitioners' understanding of the increasing prevalence of malnutrition, even among those who are overweight or obese. 28 Although morbidly-obese patients were not included in the present study, the lower BMI found in patients with a high NPS suggests that malnutrition may also be a reliable parameter to assess the degree of carotid artery stenosis.

### Limitations and strengths of the study

The study design is characterized by a series of restrictive and robust measures. Primarily, it is predicated on retrospective and single-center data. Despite this limitation, we were able to perform an analysis of the maximum number of patients by keeping the cross-sectional range wide. Consequently, despite the limitations imposed by single-center data, the substantial increase in patient numbers was instrumental in enhancing the study's statistical power. A notable constraint pertains to the absence of long-term outcomes, specifically follow-ups extending from 6 months to 1 year. Had we had the opportunity to access these data, we would have been able to clearly demonstrate the relationship involving a high NPS, which is correlated with high carotid stenosis degree, and mortality, adverse outcomes, and recurrent ischemic events. The validation of these results awaits the execution of future multicenter, prospective, and long-term follow-up studies.

In the current study, the morphological characteristics of atherosclerotic plaques (such as ulcerations, surface irregularities, and villous structures) and histopathological findings (such as intraplaque hemorrhage) were not systematically evaluated. However, it is known that these parameters may influence clinical prognosis regardless of the degree of stenosis, and they are closely related to inflammatory processes and thrombogenic potential. 29 Therefore, the failure to consider these variables constitutes one of the important limitations of the present study. Future prospective studies should include the evaluation of plaque morphology and histopathological characteristics in conjunction with clinical outcomes.

In conclusion, the current study revealed a significant correlation between carotid stenosis severity and the NPS. Clinicians can readily calculate the NPS using simple, fast, and cost-effective parameters, thereby serving as a prognostic tool to predict the severity of carotid stenosis.

**Table 1:** Demographic data, digital subtraction angiography results, and laboratory and prognostic markers

		Number (n)	Percentage (%)
Gender	Female	243	37.2
	Male	410	62.8
Hypertension		544	83.3
Diabetes mellitus		312	47.8
Coronary artery disease		342	52.4
History of coronary bypass		95	14.5
Congestive heart failure		32	4.9
Chronic obstructive pulmonary disease		82	12.6
Chronic kidney disease		90	13.8
History of cerebrovascular accident		270	41.3
Hyperlipidemia		279	42.7
Smoking		227	34.8
Carotid stenosis grade	< 70%	397	60.8
	< 50% (controls)	172	26.3
	50–69%	84	12.9
Neutrophil-to-lymphocyte ratio	Low	392	60.0
	High	261	40.0
Lymphocyte-to-monocyte ratio	Low	113	17.3
	High	540	82.7
Total cholesterol level	Low	215	32.9
	High	438	67.1
Albumin	Low	247	37.8
	High	406	62.2
Naples Prognostic Score group	0, 1 and 2	316	48.4
	3 and 5	337	51.6
Naples Prognostic Score	0	17	2.6
	1	87	13.3
	2	212	32.5
	3	214	32.8
	4	123	18.8
		**Mean ± standard deviation**	**Median (minimum–maximum)**
Age (years)		67.8 ± 9.2	69 (23–98)
Height (cm)		167.4 ± 7.7	168 (147–189)
Weight (kg)		75.9 ± 11.3	75 (30–130)
Body Mass Index (kg/m ^2^ )		27.1 ± 4.1	26.8 (9.26–48.93)
White blood cell (10 ^3^ /μL)		7.68 ± 2.0	7.5 (3.2–17.01)
Neutrophil (10 ^3^ /μL)		5.04 ± 1.7	4.8 (0.3–12)
Lymphocyte (10 ^3^ /μL)		1.87 ± 0.8	1.79 (0.3–10.8)
Monocyte (10 ^3^ /μL)		0.63 ± 0.4	0.6 (0.07–9.7)
Hemoglobin (g/dL)		13.07 ± 1.7	13.1 (1.7–17.7)
Hematocrit (%)		38.9 ± 4.6	39 (23.6–53.6)
Platelets (10 ^3^ /μL)		256.6 ± 103.4	244 (27–2015)
Creatinine (mg/dL)		0.85 ± 0.25	0.8 (0.18–2.51)
Blood urea nitrogen (mg/dL)		36.7 ± 13.2	34 (15–112)
Glomerular filtration rate (mL/minute/1.73m ^2^ )		85.1 ± 18.1	89 (25–140)
Albumin (g/dL)		3.85 ± 0.4	3.8 (0.35–4.8)
Total cholesterol (mg/dL)		187.5 ± 44.6	184 (48–385)
High-density lipoprotein (mg/dL)		44.6 ± 11.6	43 (17–153)
Low-density lipoprotein (mg/dL)		122.6 ± 33.9	121 (38–258)
Triglyceride (mg/dL)		163.6 ± 91.0	141 (8–817)
Neutrophil-to-lymphocyte ratio		3.16 ± 1.9	2.63 (0.25–16.33)
Lymphocyte-to-monocyte ratio		3.31 ± 1.8	3.06 (0.24–22)

**Table 2:** Analysis according to Naples Prognostic Score groups

		Low Naples (N = 316)	High Naples (N = 337)	*p* ^†^
		n (%)	n (%)	
Gender	Female	140 (44.3)	103 (30.6)	< 0.001*
	Male	176 (55.7)	234 (69.4)	
Hypertension		254 (80.4)	290 (86.1)	0.033*
Diabetes mellitus		162 (51.3)	150 (44.5)	0.084
Coronary artery disease		166 (52.5)	176 (52.2)	0.938
History of coronary bypass		43 (13.6)	52 (15.4)	0.509
Congestive heart failure		17 (5.4)	15 (4.5)	0.583
Chronic obstructive pulmonary disease		32 (10.1)	50 (14.8)	0.044*
Chronic kidney disease		36 (11.4)	54 (16)	0.086
History of cerebrovascular accident		131 (41.5)	139 (41.2)	0.957
Hyperlipidemia		143 (45.3)	136 (40.4)	0.206
Smoking		106 (33.5)	121 (35.9)	0.527
Carotid stenosis grade	< 70%	177 (56)	220 (65.3)	0.015*
	< 50% (controls)	100 (31.6)	72 (21.4)	0.003**
	50–69%	39 (12.4)	45 (13.4)	0.700
Neutrophil-to-lymphocyte ratio	Low	285 (90.2)	107 (31.8)	< 0.001**
	High	31 (9.8)	230 (68.2)	
Lymphocyte-to-monocyte ratio	Low	108 (34.2)	5 (1.5)	< 0.001**
	High	208 (65.8)	332 (98.5)	
Total cholesterol level	Low	177 (56)	38 (11.3)	< 0.001**
	High	139 (44)	299 (88.7)	
Albumin	Low	183 (57.9)	64 (19.0)	< 0.001**
	High	133 (42.1)	273 (81)	
		**Mean ± standard deviation (median)**	**Mean ± standard deviation (median)**	***p*** ^**‡**^
Age (years)		66.7 ± 8.9 (68)	68.9 ± 9.4 (70)	0.001**
Height (cm)		166.3 ± 7.6 (165)	168.4 ± 7.7 (170)	< 0.001**
Weight (kg)		76.0 ± 11.7 (75)	75.7 ± 10.9 (75)	0.626
Body Mass Index (kg/m ^2^ )		27.5 ± 4.3 (27.3)	26.8 ± 3.9 (26.1)	0.002**
White blood cell (10 ^3^ /μL)		7.62 ± 2.0 (7.29)	7.73 ± 2.0 (7.64)	0.279
Neutrophil (10 ^3^ /μL)		4.65 ± 1.5 (4.3)	5.39 ± 1.8 (5.2)	< 0.001**
Lymphocyte (10 ^3^ /μL)		2.24 ± 0.9 (2.1)	1.52 ± 0.5 (1.5)	< 0.001**
Monocyte (10 ^3^ /μL)		0.63 ± 0.6 (0.6)	0.62 ± 0.19 (0.6)	0.023*
Hemoglobin (g/dL)		13.2 ± 1.6 (13.2)	12.9 ± 1.8 (13)	0.024*
Hematocrit (%)		39.5 ± 4.4 (39.9)	38.3 ± 4.7 (38.5)	0.001**
Platelets (10 ^3^ /μL)		259.4 ± 71.3 (252)	253.9 ± 126.4 (240)	0.021*
Creatinine (mg/dL)		0.83 ± 0.24 (0.79)	0.87 ± 0.26 (0.81)	0.019*
Blood urea nitrogen (mg/dL)		35.1 ± 11.8 (32)	38.3 ± 14.3 (35)	0.003**
Glomerular filtration rate (mL/minute/1.73m ^2^ )		86.4 ± 18.1 (90)	83.9 ± 18.1 (88)	0.012*
Albumin (g/dL)		3.98 ± 0.4 (4)	3.72 ± 0.36 (3.8)	< 0.001**
Total cholesterol (mg/dL)		200.1 ± 44.5 (200)	175.7 ± 41.4 (171)	< 0.001**
High-density lipoprotein (mg/dL)		46.3 ± 13.0 (44)	42.9 ± 9.9 (41)	< 0.001**
Low-density lipoprotein (mg/dL)		131.7 ± 33.3 (132)	114.1 ± 32.3 (112)	< 0.001**
Triglyceride (mg/dL)		199.6 ± 99.6 (189.5)	129.9 ± 66.5 (116)	< 0.001**
Neutrophil-to-lymphocyte ratio		2.27 ± 1.1 (2.15)	3.99 ± 2.2 (3.5)	< 0.001**
Lymphocyte-to-monocyte ratio		4.09 ± 2.1 (3.8)	2.58 ± 0.9 (2.5)	< 0.001**

Notes: * *p* < 0.05; ** *p* < 0.01; ^†^ Chi-squared test; ^‡^ Mann-Whitney U test.
